# Supplementary material for: Anti-apoptotic properties of carbon monoxide in porcine oocyte during in vitro aging
Source: PeerJ. 2017 Oct 6;5:e3876. doi: 10.7717/peerj.3876 (PMC5633033; doi:10.7717/peerj.3876)
Supplement: Table S1 — Oocytes were cultivated to metaphase II and then exposed to in vitro aging in a modified M199 medium supplemented with iCORM-2 (100 µM) or iCORM-A1 (100 µM) for 24, 48 or 72 h. Control groups of oocytes were cultivated in modified M199 medium containing DMSO (in the case of iCORM-2) or distilled H2O (in the case of iCORM-A1). DMSO or distilled H2O were added in an equivalent volume as iCORM-2 or iCORM-A1 A Significant differences in the ratio of oocytes between control and iCORM groups during 24, 48 or 72 h separately are indicated with different superscripts (P < 0.05). MII, metaphase II (intact) oocytes; A, apoptotic oocytes; L, lytic oocytes; PA, parthenogenetically activated oocytes. [file peerj-05-3876-s001.docx]

| Effect of iCORM-2 on porcine oocytes during *in vitro* aging (mean±SEM) | | | | | | | | | | | | | |
| --- | --- | --- | --- | --- | --- | --- | --- | --- | --- | --- | --- | --- | --- |
|  | | DMSO | | iCORM-2 (100 µM) | | DMSO | | iCORM-2 (100 µM) | | DMSO | | iCORM-2 (100 µM) | |
|  | | 24 Hrs | | 24 Hrs | | 48 Hrs | | 48 Hrs | | 72 Hrs | | 72 Hrs | |
| MII | | 95,10±1,38^A^ | | 94,01±1,54^A^ | | 69,51±5,59^A^ | | 67,09±1,64^A^ | | 18,18±4,23^A^ | | 17,46±1,74^A^ | |
| A | | 1,10±1,45^A^ | | 1,74±0,65^A^ | | 20,46±3,96^A^ | | 21,45±1,28^A^ | | 63,64±3,74^A^ | | 60,44±2,73^A^ | |
| L | | 1,86±1,60^A^ | | 2,11±0,75^A^ | | 1,19±1,02^A^ | | 1,12±0,52^A^ | | 1,65±1,57^A^ | | 1,84±0,96^A^ | |
| PA | | 1,94±1,33^A^ | | 2,14±0,61^A^ | | 8,84±2,10^A^ | | 10,35±1,01^A^ | | 16,53±5,62^A^ | | 20,26±2,13^A^ | |
| Effect of iCORM-A1 on porcine oocytes during *in vitro* aging (mean±SEM) | | | | | | | | | | | | |  |
|  | Dest. H_2_O | | iCORM-A1 (100 µM) | | Dest. H_2_O | | iCORM-A1 (100 µM) | | Dest. H_2_O | | iCORM-A1 (100 µM) | |  |
|  | 24 Hrs | | 24 Hrs | | 48 Hrs | | 48 Hrs | | 72 Hrs | | 72 Hrs | |  |
| MII | 92,49±2,11^A^ | | 93,52±3,34^A^ | | 62,08±6,86^A^ | | 59,75±3,64^A^ | | 26,05±3,56^A^ | | 28,83±3,70^A^ | |  |
| A | 2,44±1,66^A^ | | 4,63±2,45^A^ | | 27,67±5,89^A^ | | 30,00±4,31^A^ | | 57,94±3,53^A^ | | 59,46±3,59^A^ | |  |
| L | 2,44±1,62^A^ | | 1,85±1,85^A^ | | 5,07±2,74^A^ | | 4,58±1,98^A^ | | 4,29±2,14^A^ | | 1,86±1,21^A^ | |  |
| PA | 2,63±1,74^A^ | | 0,00±0,00^A^ | | 5,19±2,73^A^ | | 5,67±2,67^A^ | | 11,73±2,91^A^ | | 9,85±3,40^A^ | |  |

The effect of inactive CORM-2 (iCORM-2) or inactive CORM-A1 (iCORM-A1) on porcine oocytes during *in vitro* aging. Oocytes were cultivated to metaphase II and then exposed to *in vitro* aging in a modified M199 medium supplemented with iCORM-2 (100 μM) or iCORM-A1 (100 μM) for 24, 48 or 72 hours. Control groups of oocytes were cultivated in modified M199 medium containing DMSO (in the case of iCORM-2) or distilled H_2_O (in the case of iCORM-A1). DMSO or distilled H_2_O were added in an equivalent volume as iCORM-2 or iCORM-A1 ^A^ Significant differences in the ratio of oocytes between control and iCORM groups during 24, 48 or 72 hours separately are indicated with different superscripts (P<0.05). *MII - metaphase II (intact) oocytes; A - apoptotic oocytes; L - lytic oocytes; PA - parthenogenetically activated oocytes.*
