# Supplementary material for: Anti-apoptotic properties of carbon monoxide in porcine oocyte during in vitro aging
Source: PeerJ. 2017 Oct 6;5:e3876. doi: 10.7717/peerj.3876 (PMC5633033; doi:10.7717/peerj.3876)
Supplement: Data S1 [file peerj-05-3876-s002.docx]

| **Relative signal intensity of HO-1 in porcine oocytes during *in vitro* aging (mean** **±SEM)** | | | | |
| --- | --- | --- | --- | --- |
|  | **0 Hrs (MII)** | **24 Hrs** | **48 Hrs** | **72 Hrs** |
| **HO-1** | 1,00±0,07^A^ | 2,20±0,16^B^ | 3,51±0,26^C^ | 6,66±1,26^D^ |

| **Relative signal intensity of HO-2 in porcine oocytes during *in vitro* aging (mean** **±SEM)** | | | | |
| --- | --- | --- | --- | --- |
|  | **0 Hrs (MII)** | **24 Hrs** | **48 Hrs** | **72 Hrs** |
| **HO-2** | 1,00±0,05^A^ | 1,33±0,08^B^ | 1,67±0,14^C^ | 3,13±0,56^D^ |

Porcine oocytes were cultivated to metaphase II (MII) or exposed to *in vitro* aging for 24, 48 or 72 hours. The results are presented as the ratio relative to the group of oocytes at metaphase II. ^A,B,C,D^ Statistically signifficant differences in the signal intensity are indicated with different superscripts (P<0.05). The Measurement of signal intensity was performed on 15 oocytes for each experimental group.
