# Supplementary material for: Anti-apoptotic properties of carbon monoxide in porcine oocyte during in vitro aging
Source: PeerJ. 2017 Oct 6;5:e3876. doi: 10.7717/peerj.3876 (PMC5633033; doi:10.7717/peerj.3876)
Supplement: Data S2 [file peerj-05-3876-s003.docx]

| Effect of heme oxygenase inhibitor (Zn-PP IX) on porcine oocytes after 24 hrs *in vitro* aging (mean±SEM) | | | | |
| --- | --- | --- | --- | --- |
|  | 0 µM | 2,5 µM | 5 µM | 25 µM |
| MII | 94,01±1,54^A^ | 93,96±0,52^A^ | 93,46±0,91^A^ | 83,41±2,09^B^ |
| A | 1,74±2,65^A^ | 1,08±1,08^A^ | 1,95±1,18^A^ | 8,54±1,93^A^ |
| L | 2,11±0,75^A^ | 2,74±1,46^A^ | 0,00±0,00^A^ | 3,17±2,33^A^ |
| PA | 2,14±0,61^A^ | 2,22±2,22^A^ | 4,59±1,97^A^ | 4,88±0,75^A^ |

| Effect of heme oxygenase inhibitor (Zn-PP IX) on porcine oocytes after 48 hrs *in vitro* aging (mean±SEM) | | | | |
| --- | --- | --- | --- | --- |
|  | 0 µM | 2,5 µM | 5 µM | 25 µM |
| MII | 67,09±1,64^A^ | 54,17±2,08^B^ | 55,99±1,65^B^ | 55,03±2,05^B^ |
| A | 21,45±2,28^A^ | 30,83±2,42^B^ | 27,0±1,14^B^ | 27,83±1,29^B^ |
| L | 1,12±1,52^A^ | 2,08±2,08^A^ | 4,65±1,35^A^ | 9,43±1,63^B^ |
| PA | 10,35±1,01^A^ | 12,92±3,97^A^ | 12,37±2,79^A^ | 7,72±2,08^A^ |

| Effect of heme oxygenase inhibitor (Zn-PP IX) on porcine oocytes after 72 hrs *in vitro* aging (mean±SEM) | | | | |
| --- | --- | --- | --- | --- |
|  | 0 µM | 2,5 µM | 5 µM | 25 µM |
| MII | 17,46±2,74^A^ | 17,38±3,58^A^ | 15,14±2,65^A^ | 12,89±2,07^A^ |
| A | 60,44±3,73^A^ | 64,27±2,53^A^ | 61,39±4,9^A^ | 67,46±3,44^A^ |
| L | 1,84±0,96^A^ | 1,32±1,32^A^ | 3,64±2,18^A^ | 2,22±2,22^A^ |
| PA | 20,26±2,13^A^ | 17,03±3,51^A^ | 19,82±3,96^A^ | 17,43±2,78^A^ |

The effect of heme oxygenase inhibitor Zn-PP IX on porcine oocytes during *in vitro* aging. Oocytes were cultivated to metaphase II and then exposed to *in vitro* aging in a modified M199 medium supplemented with Zn-PP IX at concentrations 2,5; 5; 25 μM for 24, 48 or 72 hours. Control group of oocytes were cultivated in medium containing DMSO. ^A,B^ Statistically significant differences (in rows) in the ratio of oocytes are indicated with different superscripts (P<0.05). The total number of oocytes in each experimental group was 120. *MII - metaphase II (intact) oocytes; A - apoptotic oocytes; L - lytic oocytes; PA - parthenogenetically activated oocytes.*
