# Supplementary material for: Anti-apoptotic properties of carbon monoxide in porcine oocyte during in vitro aging
Source: PeerJ. 2017 Oct 6;5:e3876. doi: 10.7717/peerj.3876 (PMC5633033; doi:10.7717/peerj.3876)
Supplement: Data S3 [file peerj-05-3876-s004.docx]

| Effect of carbon monoxide donor CORM-A1 on porcine oocytes after 24 hrs *in vitro* aging (mean±SEM) | | | | |
| --- | --- | --- | --- | --- |
|  | C | 25 µM | 50 µM | 100 µM |
| MII | 93,52±3,34^A^ | 96,67±3,33^A^ | 97,22±2,78^A^ | 94,1±2,44^A^ |
| A | 4,63±2,45^A^ | 0,00±0,00^A^ | 2,78±2,78^A^ | 4,17±4,17^A^ |
| L | 1,85±1,85^A^ | 0,00±0,00^A^ | 0,00±0,00^A^ | 0,00±0,00^A^ |
| PA | 0,00±0,00^A^ | 3,33±3,33^A^ | 0,00±0,00^A^ | 1,73±1,73^A^ |

| Effect of carbon monoxide donor CORM-A1 on porcine oocytes after 48 hrs *in vitro* aging (mean±SEM) | | | | |
| --- | --- | --- | --- | --- |
|  | C | 25 µM | 50 µM | 100 µM |
| MII | 59,75±3,64^A^ | 75,7±3,72^B^ | 72,34±3,78^B^ | 62,18±9,55^A^ |
| A | 30,00±4,31^A^ | 15,64±5,76^B^ | 19,82±4,61^B^ | 27,32±9,52^A^ |
| L | 4,58±1,98^A^ | 2,67±1,63^A^ | 0,00±0,00^A^ | 0,00±0,00^A^ |
| PA | 5,67±2,67^A^ | 6,00±4,00^A^ | 7,85±1,54^A^ | 10,5±3,43^A^ |

| Effect of carbon monoxide donor CORM-A1 on porcine oocytes after 72 hrs *in vitro* aging (mean±SEM) | | | | |
| --- | --- | --- | --- | --- |
|  | C | 25 µM | 50 µM | 100 µM |
| MII | 28,83±3,70^A^ | 24,73±3,49^A^ | 40,04±7,25^B^ | 41,33±6,04^B^ |
| A | 59,46±3,59^A^ | 60,5±6,01^A^ | 45,16±7,28^B^ | 47,21±3,15^B^ |
| L | 1,86±1,21^A^ | 0,00±0,00^A^ | 2,50±2,50^A^ | 0,00±0,00^A^ |
| PA | 9,85±3,40^A^ | 14,78±5,35^A^ | 12,3±3,35^A^ | 11,46±6,19^A^ |

The effect of carbon monoxide donor CORM-A1 on porcine oocytes during *in vitro* aging. Oocytes were cultivated to metaphase II and then exposed to *in vitro* aging in a modified M199 medium supplemented with CORM-A1 at concentrations 25; 50; 100 μM for 24, 48 or 72 hours. Control group (C) of oocytes were cultivated in medium containing iCORM-A1. ^A,B^ Statistically signifficant differences (in rows) in the ratio of oocytes are indicated with different superscripts (P<0.05). The total number of oocytes in each experimental group was 120. *MII - metaphase II (intact) oocytes; A - apoptotic oocytes; L - lytic oocytes; PA - parthenogenetically activated oocytes.*
