# Supplementary material for: Anti-apoptotic properties of carbon monoxide in porcine oocyte during in vitro aging
Source: PeerJ. 2017 Oct 6;5:e3876. doi: 10.7717/peerj.3876 (PMC5633033; doi:10.7717/peerj.3876)
Supplement: Data S4 [file peerj-05-3876-s005.docx]

| Effect of carbon monoxide donor CORM-2 on porcine oocytes after 24 hrs *in vitro* aging (mean±SEM) | | | | | |
| --- | --- | --- | --- | --- | --- |
|  | C | 5 µM | 25 µM | 50 µM | 100 µM |
| MII | 94,01±1,54^A^ | 100,00±0,00^A^ | 97,98±2,02^A^ | 98,04±1,96^A^ | 96,54±1,93^A^ |
| A | 1,74±0,65^A^ | 0,00±0,00^A^ | 1,01±1,01^A^ | 0,00±0,00^A^ | 0,00±0,00^A^ |
| L | 2,11±0,75^A^ | 0,00±0,00^A^ | 0,00±0,00^A^ | 0,00±0,00^A^ | 0,00±0,00^A^ |
| PA | 2,14±0,61^A^ | 0,00±0,00^A^ | 1,01±1,01^A^ | 1,96±1,96^A^ | 3,46±1,93^A^ |

| Effect of carbon monoxide donor CORM-2 on porcine oocytes after 48 hrs *in vitro* aging (mean±SEM) | | | | | |
| --- | --- | --- | --- | --- | --- |
|  | C | 5 µM | 25 µM | 50 µM | 100 µM |
| MII | 67,09±1,64^A^ | 77,67±0,52^B^ | 80,56±1,43^B^ | 80,13±0,94^B^ | 75,73±1,37^B^ |
| A | 21,45±1,28^A^ | 14,75±0,32^B^ | 11,54±1,46^B^ | 12,80±1,74^B^ | 13,80±1,43^B^ |
| L | 1,12±0,52^A^ | 0,00±0,00^A^ | 0,61±0,61^A^ | 0,00±0,00^A^ | 0,00±0,00^A^ |
| PA | 10,35±1,01^A^ | 7,58±1,69^A^ | 7,29±2,08^A^ | 7,07±1,56^A^ | 10,47±1,76^A^ |

| Effect of carbon monoxide donor CORM-2 on porcine oocytes after 72 hrs *in vitro* aging (mean±SEM) | | | | | |
| --- | --- | --- | --- | --- | --- |
|  | C | 5 µM | 25 µM | 50 µM | 100 µM |
| MII | 17,46±1,74^A^ | 32,58±4,99^B^ | 37,37±4,04^B^ | 39,05±2,21^B^ | 30,06±2,39^B^ |
| A | 60,44±2,73^A^ | 51,52±1,52^B^ | 43,00±3,59^B^ | 43,75±3,24^B^ | 47,35±1,45^B^ |
| L | 1,84±0,96^A^ | 0,00±0,00^A^ | 0,00±0,00^A^ | 0,88±0,88^A^ | 2,95±1,51^A^ |
| PA | 20,26±2,13^A^ | 15,91±3,50^A^ | 19,62±4,56^A^ | 16,32±3,09^A^ | 19,64±2,36^A^ |

The effect of carbon monoxide donor CORM-2 on porcine oocytes during *in vitro* aging. Oocytes were cultivated to metaphase II and then exposed to *in vitro* aging in a modified M199 medium supplemented with CORM-2 at concentrations 5; 25; 50; 100 μM for 24, 48 or 72 hours. Control group (C) of oocytes were cultivated in medium containing iCORM-2. ^A,B^ Statistically significant differences (in rows) in the ratio of oocytes are indicated with different superscripts (P<0.05). The total number of oocytes in each experimental group was 120. *MII - metaphase II (intact) oocytes; A - apoptotic oocytes; L - lytic oocytes; PA - parthenogenetically activated oocytes.*
