# Supplementary material for: Anti-apoptotic properties of carbon monoxide in porcine oocyte during in vitro aging
Source: PeerJ. 2017 Oct 6;5:e3876. doi: 10.7717/peerj.3876 (PMC5633033; doi:10.7717/peerj.3876)
Supplement: Data S5 [file peerj-05-3876-s006.docx]

| **Effect of carbon monoxide donor CORM-A1 on the expression of activated caspase-3 in porcine oocytes after 24 hrs *in vitro* aging (mean±SEM)** | | | | |
| --- | --- | --- | --- | --- |
|  | C | 25 µM | 50 µM | 100 µM |
| CAS-3 | 100,00±6,01^A^ | 51,55±6,15^B^ | 59,15±5,88^B^ | 83,48±5,24^C^ |

| **Effect of carbon monoxide donor CORM-A1 on the expression of activated caspase-3 in porcine oocytes after 48 hrs *in vitro* aging (mean±SEM)** | | | | |
| --- | --- | --- | --- | --- |
|  | C | 25 µM | 50 µM | 100 µM |
| CAS-3 | 65,63±10,34^A^ | 48,92±4,85^B^ | 42,11±4,53^B^ | 65,00±3,54^A^ |

| **Effect of carbon monoxide donor CORM-A1 on the expression of activated caspase-3 in porcine oocytes after 72 hrs *in vitro* aging (mean±SEM)** | | | | |
| --- | --- | --- | --- | --- |
|  | C | 25 µM | 50 µM | 100 µM |
| CAS-3 | 69,03±7,62^A^ | 55,56±5,16^B^ | 46,11±8,72^B^ | 62,41±3,92^A^ |

The effect of carbon monoxide donor CORM-A1 on the expression of activated caspase-3 (CAS-3). . Oocytes were cultivated to metaphase II and then exposed to *in vitro* aging in a modified M199 medium supplemented with CORM-A1 at concentrations 25; 50; 100 μM for 24, 48 or 72 hours. Control group (C) of oocytes were cultivated in medium containing iCORM-A1. The results are presented as the relative ratio to the control group (0 µM CORM-A1) of oocytes aged 24 hours. ^A,B,C^ Statistically significant differences in the level of expression of the activated CAS-3 between control group a CORM-A1 groups are indicated with different superscripts (P<0.05). The Measurement of signal intensity was performed on 15 oocytes for each experimental group.
