# Supplementary material for: Anti-apoptotic properties of carbon monoxide in porcine oocyte during in vitro aging
Source: PeerJ. 2017 Oct 6;5:e3876. doi: 10.7717/peerj.3876 (PMC5633033; doi:10.7717/peerj.3876)
Supplement: Data S6 [file peerj-05-3876-s007.docx]

| **Effect of carbon monoxide donor CORM-2 on the expression of activated caspase-3 in porcine oocytes after 24 hrs *in vitro* aging (mean±SEM)** | | | | |
| --- | --- | --- | --- | --- |
|  | C | 25 µM | 50 µM | 100 µM |
| CAS-3 | 100,00±8,37^A^ | 36,29±4,48^B^ | 44,44±2,66^B^ | 38,08±2,12^B^ |

| **Effect of carbon monoxide donor CORM-2 on the expression of activated caspase-3 in porcine oocytes after 48 hrs *in vitro* aging (mean±SEM)** | | | | |
| --- | --- | --- | --- | --- |
|  | C | 25 µM | 50 µM | 100 µM |
| CAS-3 | 35,33±2,88^A^ | 18,72±2,57^B^ | 13,07±1,88^B^ | 22,35±1,83^B^ |

| **Effect of carbon monoxide donor CORM-2 on the expression of activated caspase-3 in porcine oocytes after 72 hrs *in vitro* aging (mean±SEM)** | | | | |
| --- | --- | --- | --- | --- |
|  | C | 25 µM | 50 µM | 100 µM |
| CAS-3 | 24,86±5,24^A^ | 13,63±2,55^B^ | 10,8±3,83^B^ | 14,08±1,97^B^ |

The effect of carbon monoxide donor CORM-2 on the expression of activated caspase-3 (CAS-3). . Oocytes were cultivated to metaphase II and then exposed to *in vitro* aging in a modified M199 medium supplemented with CORM-2 at concentrations 25; 50; 100 μM for 24, 48 or 72 hours. Control group (C) of oocytes were cultivated in medium containing iCORM-2. The results are presented as the relative ratio to the control group (0 µM CORM-2) of oocytes aged 24 hours. ^A,B^ Statistically significant differences in the level of expression of the activated CAS-3 between control group a CORM-2 groups are indicated with different superscripts (P<0.05). The Measurement of signal intensity was performed on 15 oocytes for each experimental group.
